# Gendered association between life‐course social mobility and cognitive function among middle‐aged and older adults in India

**DOI:** 10.1002/alz.085910

**Published:** 2025-01-09

**Authors:** Soohyeon Ko, Rockli Kim

**Affiliations:** ^1^ Korea University, Seongbuk‐gu Korea, Republic of (South)

## Abstract

**Background:**

In rapidly developing countries like India, individuals undergo fluctuations in social status throughout their lives. However, there have been few investigations of the link between life course socioeconomic status and cognitive function in late middle age, particularly regarding whether upward and downward mobility exert symmetric effects. Furthermore, limited attention has been directed toward understanding gender heterogeneity within this association.

**Method:**

This study utilized data from the Longitudinal Aging Study in India 2017‐2018, including 61,257 individuals aged 45 years or above. Based on financial status of family during childhood and monthly per capita expenditure in later life, life course social mobility was classified as “consistently high,” “upward mobility,” downward mobility,” and “consistently low.” Cognitive function was evaluated using the composite cognitive index across five domains (memory, orientation, arithmetic function, executive function, and object naming). Multivariable regression and interaction analysis were employed for estimating associations, with a focus on gender differences.

**Result:**

Participants, with a mean age of 60.13 years, consisted of 45.93% men and 54.07% women. Overall, compared to those who maintained consistently high status, individuals remaining consistently low were associated with the lowest cognitive function (b = ‐1.48, 95% CI = ‐1.60, ‐1.35), followed by those undergoing downward mobility (b = ‐0.86, 95% CI = ‐0.98, ‐0.74) and upward mobility (b = ‐0.76, 95% CI = ‐0.87, ‐0.64). Significantly, the difference between upward and downward mobility varied by gender (p = 0.016). Among men, upward and downward mobility had asymmetric associations, with upward mobility being associated with a higher cognitive function than downward mobility (b = 0.30, 95% CI = 0.11, 0.49), whereas no significant difference was found for women (b = ‐0.03, 95% CI = ‐0.21, 0.16).

**Conclusion:**

This study revealed notable disparities in cognitive function based on life course socioeconomic status. Furthermore, the association of upward and downward mobility on cognitive function exhibited gender‐specific differences. While for men, childhood disadvantage in cognitive function could be offset by upward mobility in adulthood, women experiencing low status at either temporal point in childhood or adulthood displayed lower cognitive function. These findings underscore the importance of tailored interventions, considering life trajectories of social status and gender, to address cognitive health disparities in rapidly evolving economies like India.

